# Reference Intervals for Serum Protein Electrophoresis in the European Bison (*Bison bonasus*): A Comparison of Agarose Gel Electrophoresis and Capillary Zone Electrophoresis

**DOI:** 10.3390/vetsci13070644

**Published:** 2026-06-30

**Authors:** Anna Didkowska, Victor Martín Santander, Daniel Klich, Michal Skibniewski, Katarzyna Matusik, Marlena Wojciechowska, Wanda Olech, Krysztof Anusz, Diana Marteles-Aragüés, Sergio Villanueva-Saz, Antonio Fernández

**Affiliations:** 1 Department of Food Hygiene and Public Health Protection, Institute of Veterinary Medicine, Warsaw University of Life Sciences (SGGW), Nowoursynowska 166, 02-787 Warsaw, Poland; katarzyna_matusik1@sggw.edu.pl (K.M.); krzysztof_anusz@sggw.edu.pl (K.A.); 2 Department of Animal Pathology, Instituto Agroalimentario de Aragón-IA2 (Universidad de Zaragoza-CITA), Veterinary Faculty, University of Zaragoza, 50013 Zaragoza, Spain; victor.martin1990@gmail.com (V.M.S.); dmartelesaragues@unizar.es (D.M.-A.); afmedica@unizar.es (A.F.); 3 Clinical Immunology Laboratory, Veterinary Faculty, University of Zaragoza, 50013 Zaragoza, Spain; 4 Department of Animal Genetics and Conservation, Warsaw University of Life Sciences (SGGW), Ciszewskiego 8, 02-786 Warsaw, Poland; daniel_klich@sggw.edu.pl (D.K.); marlena_wojciechowska@sggw.edu.pl (M.W.); wanda_olech@sggw.edu.pl (W.O.); 5 Department of Morphological Sciences, Faculty of Veterinary Medicine, Warsaw University of Life Sciences, 02-776 Warsaw, Poland; michal_skibniewski@sggw.edu.pl

**Keywords:** AGE, CZE, European bison, reference intervals, serum proteins

## Abstract

Knowledge of the reference intervals for serum proteins of the European bison (*Bison bonasus*) can be of great help in the diagnosis of infectious and inflammatory diseases, which are very common in these animals; however, reference intervals for this species are unknown. A total of 131 serum samples were obtained from the European bison from different locations in Poland. Agarose gel electrophoresis (AGE) and capillary zone electrophoresis (CZE) were used to separate and identify protein fractions. In all sera, six fractions were identified: albumin, α1, α2, β1-, β2-, and γ-globulins by both analytical methods. Reference intervals (90% CI) were determined. Comparisons were made to find significant differences based on the sex, age or lifestyle of the animals. Concordance between the methods was assessed using Passing–Bablok regression, Bland–Altman analysis, and Lin’s concordance correlation. A higher percentage of α1-globulins was found in the females than in the males. Captive bison had significantly lower γ-globulin levels compared to free-living bison. The γ-globulin levels increased with the age of the animals. The Bland–Altman analysis demonstrated a systematic bias indicating no good agreement between methods, and only the values for albumin, β1-, β2-, γ-globulins, and total globulins were considered acceptable according to Lin’s concordance correlation. Both methods are adequate for identifying serum proteins, but they are not interchangeable, necessitating the use of separate reference intervals for each method.

## Simple Summary

The European bison (*Bison bonasus*) is a species that came close to extinction in the last century. Although conservation measures have prevented its disappearance, infectious diseases continue to threaten its recovery. Blood protein electrophoresis is a widely used laboratory technique in veterinary medicine and serves as an important diagnostic tool for inflammatory and infectious diseases. However, reference intervals necessary for accurate interpretation of results are still lacking. Serum samples were collected from 131 bison of both sexes, representing different age groups and locations across Poland. Agarose gel electrophoresis (AGE) and capillary zone electrophoresis (CZE) were employed to separate and identify protein fractions. Because electrophoretic profiles obtained using different techniques are not directly comparable, it is essential to establish technique-specific reference intervals. In all serum samples, six protein fractions were identified by both analytical methods: albumin, α1-, α2-, β1-, β2-, and γ-globulins. Statistically significant differences were observed according to age, sex, and lifestyle. Although both methods proved valid, concordance and correlation analyses demonstrated that they are not interchangeable; therefore, separate reference intervals are required for each technique. These findings will support veterinarians working in zoos and wildlife reserves in the diagnosis of infectious and inflammatory diseases in European bison.

## 1. Introduction

The European bison (*Bison bonasus*), also named wisent, is a threatened species in Europe, but its population has fortunately been increasing in recent years due to conservation efforts, particularly in Poland [1]. In the conservation of wild species, it is important to have clinical laboratory data to improve the diagnosis of animal diseases. Among conservation practices, surveillance and control of animal diseases are crucial. The lack of reference values can make it difficult to diagnose many diseases. Recently, Didkowska et al. [2] published biochemical and haematological blood parameters as tools for diagnosing disease in captive animals and for assessing the health status of wildlife populations of this species. Increasing population density and a limited gene pool elevate the risk of infectious diseases in these animals. To assess health status, particularly in relation to infectious processes, the analysis of serum proteins is highly useful for diagnosing diseases [3,4], and acute-phase proteins (APPs) play a significant role in evaluating the general health of wildlife [5]. Pomorska-Mól et al. [6] demonstrated that concentrations of haptoglobin and serum amyloid A were higher in culled European bison than in clinically healthy individuals. Many of these APPs are found in the alpha and beta fractions of serum protein electrophoresis [3,4].

Additionally, Pomorska-Mól et al. [7] observed age-related changes in total immunoglobulin (Ig) and IgG concentrations, with higher concentrations in older European bison. IgG resides in the γ-globulin region of serum proteins [3,4]. Serum protein electrophoresis is a widely used laboratory method in veterinary medicine and is valuable for diagnosing serum protein disorders such as polyclonal gammopathy, hypoalbuminemia, and hypoglobulinemia. Electrophoresis of serum proteins has proven to be effective in the early detection of subclinical diseases, providing predictive insights into disease development, such as leishmaniosis in cats [8] and domestic ferrets [9], and in the diagnosis of neonatal septicaemia in foals [10].

One challenge in applying laboratory variables to wild animals is the lack of reference intervals, particularly for serum proteins separated by electrophoresis. Currently, reference intervals for serum protein electrophoresis in European bison are lacking, but they are essential for in-depth studies on health status and immunity. Wolk and Józefczak [11] analysed serum proteins from 57 free-ranging European bison across different age groups but did not establish reference intervals. For other wild species, reference intervals have recently been published, such as for the European mink (*Mustela lutreola*) [12], bottlenose dolphins (*Tursiops truncatus*) [13], and bald eagles (*Haliaeetus leucocephalus*) [14].

Serum protein electrophoresis is a useful, inexpensive tool available to all veterinary clinical laboratories for the identification of infections and inflammations in animals [3]. Several techniques are available for analysing serum proteins, including cellulose acetate electrophoresis (CAE), agarose gel electrophoresis (AGE), and capillary zone electrophoresis (CZE). CZE offers advantages over other methods, such as higher separation efficiency and shorter assay times. The CZE technique also allows for the separation of more protein fractions. However, electrophoretic profiles obtained using different techniques are not directly comparable. Studies in various wildlife species, such as bottlenose dolphins [13] and European mink [12], have shown that CZE and AGE techniques are not equivalent, necessitating the calculation of reference intervals for each technique. Several authors have pointed out the advantage of CZE over AGE because it has better resolution of protein fractions; however, because the profiles obtained differ between the two techniques, the correct interpretation of the fractions can be difficult [15,16].

The aim of this study was to establish reference intervals for serum proteins in European bison using two electrophoresis techniques, AGE and CZE. Reference intervals were determined according to the 2008 Clinical Laboratory Standards Institute (CLSI) guidelines [17]. A secondary objective was to assess the concordance between the two serum protein electrophoresis methods in European bison serum and to evaluate the impact of sex, age, and type of maintenance on electrophoresis results.

## 2. Materials and Methods

### 2.1. Sampling of Healthy European Bison

From 2017 to 2023, 131 healthy European bison were sampled during immobilization (77 females, 54 males). The immobilization was carried out for specific management or conservation purposes in the context of the relocation of European bison. This immobilization was opportunistic and only applied to selected individuals. During the immobilization, biological material was collected for monitoring the animals’ health. The animals were clinically healthy based on clinical examination by the veterinarian and were tested for tuberculosis, bovine leukaemia and brucellosis. These tests were performed with blood sent to the specialized reference laboratory at the National Veterinary Research Institute in Puławy (Poland) for analysis. The animals showed no skin lesions consistent with mange or other external parasites. Body condition was considered optimal if it was above 3 on a scale of 5, based on a visual assessment from the side and rear of the animal. Only clinically healthy animals with negative disease results were chosen for this study. The mean age of the animals was 6.7 ± 4.1 years. Animals were from captive (*n* = 85) or free-ranging (*n* = 46) herds from different regions of Poland. Most of the samples were collected as part of the ‘Complex project of European bison conservation by State Forests’ (Contract No. OR. 271.3.10.2017).

### 2.2. Serum Protein Electrophoresis

Blood samples were obtained from the jugular vein or caudal vein using vacuum tubes. The blood was allowed to clot by leaving it undisturbed at room temperature for 60–90 min, and the clot was removed by centrifugation at 1500× *g* for 10 min. Total protein was measured by the biuret method using the Cobas^®^ 8000 Analyzer Series Module C701 (Roche Diagnostics, Mannheim, Germany).

Two methods were used for the separation of blood proteins: agarose gel electrophoresis (AGE) and capillary zone electrophoresis (CZE). Serum protein electrophoresis was performed by AGE (Hydragel Kit 1-2; Sebia, Issy-les-Moulineaux, France). The AGE procedure was conducted according to the manufacturer’s instructions. The electrophoretic curve for each sample was displayed and read with a GELSCAN TM densitometry system. Protein fractions were determined as a percentage of optical absorbance, and the absolute concentration (g/dL) was automatically calculated from the total serum protein concentration using a spectrophotometer. Albumin-to-globulin (A:G) ratios were also calculated.

CZE was performed at Laboklin GmbH & Co. KG (Bad Kissingen, Germany) using Sebia Capillarys 3 OCTA (Sebia, Lissex, Evry Cedex, France). The protein fractions were analysed using the veterinary mode of Sebia software. Electropherograms can be visually interpreted to screen for pattern abnormalities. Direct detection provides accurate relative quantification of individual protein fractions.

### 2.3. Statistical Analysis

The main objective of this research was to provide the reference intervals for serum proteins obtained with two electrophoresis methods, AGE and CZE. Statistical analysis was conducted using MedCalc^®^ Statistical Software version 22.021 (MedCalc Software Ltd., Ostend, Belgium; https://www.medcalc.org, accessed on 27 June 2026; 2024). Since the sample size was ≥120, reference intervals (RIs) were determined using a nonparametric method with 90% confidence intervals (CIs), following the guidelines of the Clinical and Laboratory Standards Institute (CLSI) [17]. Outliers were identified using Tukey’s test and excluded from further analysis. A secondary objective was to determine if there were statistically significant differences for the variables age, sex, or whether the bison were free-living or captive. The Shapiro–Wilk test was used to determine if the samples were normally distributed. Not all samples followed a normal distribution. Nonparametric tests, namely the Mann–Whitney U test and the Kruskal–Wallis test, were used for samples with a non-normal distribution. We checked whether there were statistically significant differences for each of the electrophoretic methods separately for sex (male and female), and living conditions (captive vs. free-ranging). Unpaired comparisons across different age groups (calves < 1 year old, young 2–3 years old, adults > 4 years old) were analysed using ANOVA, followed by Duncan’s multiple range test as a post-hoc analysis.

Concordance between the two electrophoresis methods was evaluated using Passing–Bablok regression analysis and the Bland–Altman method. Lin’s concordance correlation coefficient (CCC) was used to assess the strength of the concordance between measurements. Statistical significance was defined as *p* ≤ 0.05.

## 3. Results

**Figure 1:**
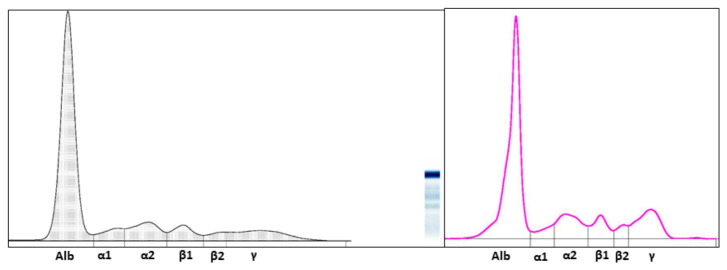
Comparison of the same European bison electrophoretic tracings produced by the AGE (**left**) and CZE (**right**) methods.

The reference intervals for the protein fractions of European bison, expressed as both percentages and concentrations (g/dL), are provided for the AGE method (Table 1) and the CZE method (Table 2). In the CZE method, the protein peaks were narrower compared to the AGE profiles. Both methods identified six distinct protein fractions using the corresponding software: albumin, α1-globulin, α2-globulin, β1-globulin, β2-globulin, and γ-globulins (Figure 1). The differences between males and females are presented in Table S1. No statistically significant differences (*p* > 0.05) were observed between sexes for the total protein and protein fractions analysed using the AGE method. However, when using the CZE method, a statistically significant difference (*p* < 0.05) was identified: the percentage of α1-globulins was higher in females than in males.

Table S2 presents the differences between captive and free-living bison. Using the AGE method, higher percentages of α1- and α2-globulins (*p* < 0.05) were observed in captive animals, while γ-globulin levels were lower in these individuals. However, when using the CZE method, captive bison had significantly higher γ-globulin levels compared to free-living bison.

Table S3 presents the differences in blood protein fractions across age groups. The most notable differences in both methods were observed in the percentage of γ-globulins, which were significantly lower in calves compared to other age groups (*p* < 0.001). Significant differences were also found in the levels of α1- and α2-globulins using both methods. When protein fractions were analysed with the CZE method, significantly lower percentages were found compared to calves and young animals for α1-, α2-, and γ-globulins.

Tables S4 and S5 present the results of the Passing–Bablok regression analysis and Bland–Altman analysis comparing the AGE and CZE electrophoresis methods. The Passing–Bablok regression analysis revealed a constant bias for α2-, β2-, γ-globulins, total globulins and the A:G ratio when data were expressed as percentages, and for albumin, α2-, and β2-globulins when expressed as concentrations. Proportional bias between the two methods was identified for all protein fractions, except for β1-globulin, regardless of whether the results were expressed as percentages or concentrations.

The Bland–Altman analysis showed that there was a systematic bias between both methods, which was positive or negative for the standard AGE method, expressed both as a percentage and as a concentration in g/dL. This systematic bias between the two methods was supported by the fact that none of the protein fractions had a value of 0 within the confidence interval (CI) of the arithmetic mean, indicating poor agreement between the methods.

Table S6 shows Lin’s concordance correlation between the AGE and CZE methods. The highest concordance was found when data were expressed as concentrations, showing good concordance for albumin, β1-, β2-, γ-globulins, and total globulins (concordance correlation > 0.5).

## 4. Discussion

In this study, 131 serum samples from apparently healthy European bison were collected across different regions of Poland. This provides a strong basis for establishing new reference intervals for various analytes [17]. These values provide a good basis for clinical veterinarians and wildlife managers in the diagnosis of diseases, mainly infectious ones, and have been used in other wild species such as the European mink [12]. A secondary objective was to explore whether there were statistically significant differences between sexes, different age groups, and whether the European bison came from the wild or were captive. The analyses were performed independently and do not account for potential confounding variables, and therefore, these findings should be interpreted cautiously and considered hypothesis-generating rather than definitive.

Wolk and Józefczak [11] reported TP levels in European bison, noting lower values in females (6.34 ± 0.51 g/dL) compared to males (7.52 ± 0.5 g/dL). In our study, no significant differences in TP levels were observed between sexes, free-ranging versus captive animals, or across age groups. While TP concentrations in adult animals were slightly higher than in younger animals, as reported by Peinado et al. [18], these differences were not significant. Typically, TP levels increase with age, primarily due to rising globulin levels, particularly γ-globulins, which reflect the animal’s exposure to infections or immunizations over its lifespan [3,4].

No reference values have been previously established for European bison blood proteins analysed using AGE or CZE. Therefore, alternative approaches include the transference and validation of reference intervals from other species, multicentre reference intervals, and subject-based reference intervals [18]. Both techniques identified six protein fractions, although the peak sizes were slightly smaller with the CZE method.

Wolk and Józefczak [11] found significant differences between sexes, where males exhibited higher levels of α1- and γ-globulins compared to females. The authors also noted differences between free-ranging and captive animals. Hormonal effects can influence serum proteins, with catabolic or anabolic changes affecting protein levels; for instance, albumin levels decrease while globulin levels increase during pregnancy [3]. In the present study, the hormonal status or pregnancy of the females was unknown, and no significant effect of sex on serum protein electrophoresis was observed.

Elevated levels of γ-globulins were detected in free-ranging animals using both electrophoresis methods. In Poland, infectious diseases are common among free-ranging European bison, particularly liver infections caused by *Fasciola hepatica*, necrotic balanoposthitis in males, and pneumonia [5,19]. A higher concentration of APP was also observed in 47 culled European bison in Poland, reflecting poor health conditions [6]. Some diseases, such as *Neospora caninum* infections, which may be transmitted by local wolves, are more prevalent in free-ranging herds than in captive ones [19].

In this study, the European bison were clinically healthy at the time of blood sampling. However, significant differences in protein levels, particularly γ-globulins, were noted as the animals aged, and this was consistent across both electrophoresis techniques. Peinado et al. [18], using CAE to analyse serum from 20 European bison, found no significant differences between young (*n* = 4) and adult (*n* = 16) animals in any protein fraction. This lack of significant findings may have been due to the small sample size. Similarly, increases in γ-globulins with age were observed by Wolk and Józefczak [11] in European bison. Pomorska-Mól et al. [7] also found that total immunoglobulin levels, particularly IgG, increased with age in European bison. Immunoglobulins are primarily concentrated in the γ-globulin fraction [3,4].

AGE and CZE produce similar electropherograms, and both methods can be effectively used to diagnose dysproteinemias. The differences observed in peak profiles arise from the software used to identify the inflection points of the protein fractions, which results in narrower peaks and the generation of more subpeaks with the CZE method. This can pose challenges in identifying protein fractions, particularly at the transitions between α- and β-globulins and between β- and γ-globulins [13,20].

Passing–Bablok regression and Bland–Altman analyses showed that the AGE and CZE methods for protein electrophoresis are not equivalent. This conclusion was similarly reached in a recent study on European mink serum proteins [12]. Both methods identified six fractions: albumin, α1, α2, β1, β2, and γ-globulins, consistent with findings in small ruminants [21]. However, Peinado et al. [18] identified only a single β-globulin fraction in five species of wild ruminants kept in zoos, and Wolk and Józefczak [11] reported similar findings in European bison. The lack of concordance between AGE and CZE highlights the importance of method-specific reference values for accurate interpretation of results. If the two methods do not produce identical results, even within the limits of combined imprecision, they cannot be used interchangeably and distinct reference intervals are required [22]. Both a constant bias and proportional bias were observed for all electrophoresis fractions except α1 and β1-globulins, whether assessed by percentage or concentration. Furthermore, the concordance correlation coefficient was deemed unacceptable for most fractions, reinforcing the need to establish method-specific reference intervals for serum protein electrophoresis in European bison.

## 5. Conclusions

This study establishes reference intervals for serum protein electrophoresis using both AGE and CZE methods in European bison. The results indicate that the two methods are not interchangeable, necessitating the use of separate reference intervals for each method. The primary finding was an age-related increase in γ-globulin levels, reflecting greater exposure to pathogens and the resultant stimulation of globulin production. Captive animals had higher α1- and α2-globulin values than free-ranging animals, but γ-globulin values were lower in captive animals. These reference intervals can be useful in the diagnosis of infectious diseases in European bison, especially in chronic conditions, in which notable alterations in γ-globulin levels are found. Finally, the collected sample size (*n* = 131) provides a robust foundation for future research on changes in blood proteins associated with specific inflammatory diseases or infections in European bison.

## Figures and Tables

**Table 1:** Reference intervals of protein fractions (in percentage and g/dL) obtained by AGE electrophoresis in European bison (*Bison bonasus*) serum. Total protein values (g/dL) were obtained by the biuret method.

Analyte	*n*	Mean	SD	Median	CV	Min	Max	RI	LRL 90% CI	URL 90% CI
Total proteins (g/dL)	130	6.4	1.21	6.28	20.5	3.13	10.7	4.1–9.7	3.13–4.71	8.5–10.7
Albumin (%)	131	58.9	5.52	59.2	9.4	43.2	69.2	47.2–69	43.2–50.7	67.2–69.2
Albumin (g/dL)	131	3.79	0.86	3.66	22.6	1.91	7.15	2.2–5.28	1.9–2.6	5.1–6.7
α1-globulins (%)	131	6.3	1.55	6.1	24.6	2.2	11.2	3.9–9.7	2.2–4.2	9.3–11.2
α1-globulins (g/dL)	131	0.4	0.11	0.38	27.2	0.11	0.81	0.17–0.62	0.11–0.22	0.58–0.81
α2-globulins (%)	131	10.1	1.62	10.2	16.2	6.4	14.1	6.5–12.8	6.4–6.9	12.4–14.1
α2-globulins (g/dL)	131	0.64	0.14	0.64	21.8	0.28	1.19	0.39–0.93	0.28–0.43	0.84–1.02
β1-globulins (%)	130	6.9	1.03	6.8	17.5	4.9	10.9	5.1–9.7	4.9–5.4	8.8–11.3
β1-globulins (g/dL)	129	0.44	0.09	0.43	21.2	0.21	0.72	0.28–0.66	0.22–0.3	0.59–0.72
β2-globulinas (%)	130	5.3	1.27	5.2	23.7	2.9	10.6	3.33–8.47	2.9–3.5	7.7–10.6
β2-globulinas (g/dL)	126	0.33	0.09	0.32	29.5	0.14	0.65	017–0.73	0.14–0.2	0.57–0.77
γ-globulins (%)	131	12.3	3.1	11.9	25.1	3.3	21.9	6.8–19.9	3.3–8.2	18.1–22
γ-globulins (g/dL)	130	0.79	0.28	0.73	35.8	0.19	1.82	0.44–1.43	0.37–0.49	1.16–1.56
Total globulins (%)	131	40.9	5.4	40.5	13.3	30.8	56.8	31–52.8	30.8–32.8	51.3–56.8
Total globulins (g/dL)	131	2.62	0.64	2.53	24.7	1.22	5.35	1.82–3.82	1.72–1.9	3.43–4.56
A:G ratio	131	1.48	0.33	1.45	22.2	0.76	2.25	0.99–2.1	0.89–1.05	1.97–2.23

CV = coefficient of variation; RI = reference intervals; SD = standard deviation; LRL 90% CI = lower reference limits, 90% confidence interval; URL 90% CI = upper reference limits, 90% confidence interval (the Raw data can be found in File S1).

**Table 2:** Reference intervals of protein fractions (in percentage and g/dL) obtained by CZE electrophoresis in European bison (*Bison bonasus*) serum.

Analyte	*n*	Mean	SD	Median	CV	Min	Max	RI	LRL 90% CI	URL 90% CI
Albumin (%)	131	51.7	5.04	51.6	9.9	33.6	60.6	39.8–59.7	36.4–42.8	57.8–60.6
Albumin (g/dL)	130	3.25	0.63	3.21	19.4	1.68	5.22	2.04–4.9	1.7–2.28	4.34–5.22
α1-globulins (%)	131	3.8	1.04	3.9	27.2	1.2	7.9	1.9–5.9	1.2–2.1	5.2–7.9
α1-globulins (g/dL)	131	0.24	0.06	0.24	29.3	0.09	0.44	0.12–0.39	0.09–0.12	0.34–0.44
α2-globulins (%)	131	15.4	3	15.1	19.6	6.9	23.4	9.8–21.9	6.9–10.8	10.4–23.4
α2-globulins (g/dL)	131	0.99	0.27	0.96	27.5	0.37	1.7	0.44–1.6	0.37–0.59	1.46–1.7
β1-globulins (%)	131	6.5	1.05	6.5	16.2	4.4	11.2	4.8–9	4.4–5.1	7.8–9.3
β1-globulins (g/dL)	130	0.41	0.09	0.4	22.4	0.19	0.76	0.22–0.63	0.19–0.28	0.57–0.66
β2-globulins (%)	128	4.57	0.7	4.5	15.6	3.3	6.8	3.3–6.4	3.3–3.5	6–6.8
β2-globulins (g/dL)	128	0.29	0.09	0.28	30.4	0.13	0.67	0.17–0.46	0.14–0.19	0.44–0.56
γ-globulins (%)	131	18.7	3.5	17.9	18.7	12.3	30.7	13.7–25.9	12.8–14.4	23.8–27.8
γ-globulins (g/dL)	130	1.21	0.4	1.14	32.5	0.57	2.62	0.67–2.28	0.57–0.73	1.85–2.61
Total globulins (%)	131	48.7	5.4	48	11.2	26.5	66.4	40.3–60.1	39.3–42.1	57.4–63.6
Total globulins (g/dL)	131	3.15	0.79	3.05	23.7	1.45	6.34	2.07–4.68	1.87–2.25	4.2–5.57
A:G ratio	131	1.07	0.22	1.07	20.6	0.51	1.95	0.72–1.39	0.65–0.79	1.36–1.53

CV = coefficient of variation; RI = reference intervals; SD = standard deviation; LRL 90% CI = lower reference limits, 90% confidence interval; URL 90% CI = upper reference limits, 90% confidence interval (the Raw data can be found in File S1).

## Data Availability

The data presented in this study are available on request from the corresponding author. The data are not publicly available due to privacy restrictions.

## Supplementary Materials

The following supporting information can be downloaded at: https://www.mdpi.com/article/10.3390/vetsci13070644/s1. Table S1: Differences between bison’s female and male for total protein and protein fractions for AGE and CZE in European bison (*Bison bonasus*) serum. Table S2: Differences between bison’s captive and free-ranging for total protein and protein fractions for AGE and CZE in European bison (*Bison bonasus*) serum. Table S3: Differences between age for total protein and protein fractions for AGE and CZE in European bison (*Bison bonasus*). Table S4. Analysis of concordance between methods of electrophoresis for European bison (*Bison bonasus*) serum when data are expressed in percentage. Intercept, slope and residual standard deviation of Passing-Bablok regression as well as bias, lower and upper limit of agreement from de Bland-Altman analysis and their 95% confidence intervals (95% CI) from the comparisons between the AGE and CZE electrophoresis. Table S5. Analysis of concordance between methods of electrophoresis for European bison (*Bison bonasus*) serum when data are expressed in g/dL. Intercept, slope and residual standard deviation of Passing-Bablok regression as well as bias, lower and upper limit of agreement from de Bland-Altman analysis and their 95% confidence intervals (95% CI) from the comparisons between the AGE and CZE electrophoresis. Table S6. Concordance correlation analysis between AGE and CZE electrophoresis. Lin’s concordance correlation coefficients (r_ccc_) with 95% confidence intervals (95% CI), precision (Pearson p) and accuracy (bias correction factor, C_b_) are reported; File S1: Raw data for Table 1 and Table 2.

## Abbreviations

The following abbreviations are used in this manuscript:

AGE	Agarose gel electrophoresis
ANOVA	Analysis of variance
CAE	Cellulose acetate electrophoresis
CCC	Concordance correlation coefficient
CI	Confidence intervals
CLSI	Clinical Laboratory Standards Institute
CZE	Capillary zone electrophoresis
IUCN	International Union for Conservation of Nature
RI	Reference intervals
TP	Total protein
