# Supplementary material for: Reference Intervals for Serum Protein Electrophoresis in the European Bison (Bison bonasus): A Comparison of Agarose Gel Electrophoresis and Capillary Zone Electrophoresis
Source: Vet Sci. 2026 Jun 30;13(7):644. doi: 10.3390/vetsci13070644 (PMC13418574; doi:10.3390/vetsci13070644)
Supplement: Supplementary file 1 [file vetsci-13-00644-s001.zip › Table S1.pdf]

Table S1: Differences between bison's female and male for total protein and protein fractions for AGE and CZE in European bison (*Bison bonasus*) serum. Females=77, males=54

	AGE			CZE		
**Analyte**	**Female**	**Male**	**P value**	**Female**	**Male**	**P value**
Total proteins (g/dl)	6.4±1.4	6.4±1.2	0.721	-	-	-
Albumin (%)	58.6±5.5	59.4±5.6	0.408	50.8±5	51.5±5.1	0.359
Albumin (g/dl)	3.8±0.89	3.8±0.8	0.439	3.2±0.63	3.31±0.62	0.434
α1-globulins (%)	6.34±1.6	6.2±1.5	0.721	3.9±1	3.45±1.05	0.047
α1-globulins (g/dl)	0.4±0.11	0.4±0.11	0.922	0.24±0.06	0.22±0.07	0.069
α2-globulins (%)	10±1.7	10.1±1.5	0.766	15±3.1	16.8±2.9	0.138
α2-globulins (g/dl)	0.64±0.15	0.65±0.1	0.782	0.96±0.27	1.02±0.28	0.207
β1-globulins (%)	6.8±1.33	6.8±1	0.868	6.6±1	6.5±1	0.353
β1-globulins (g/dl)	0.43±0.09	0.44±0.1	0.844	0.42±0.09	0.41±0.09	0.669
β2-globulins (%)	5.4±1.3	5.3±1.2	0.677	4.6±0.73	4.6±0.69	0.571
β2-globulins (g/dl)	0.33±0.1	0.34±0.1	0.512	0.3±0.09	0.3±0.08	0.861
γ-globulins (%)	12.5±2.8	11.9±3.5	0.161	19±3.6	18.3±3.3	0.253
γ-globulins (g/dl)	0.81±0.28	0.8±0.3	0.511	1.22±0.41	1.19±0.36	0.798
Total globulins (%)	41.3±5.4	40.4±5.6	0.383	48.8±5.8	48.6±5.1	0.592
Total globulins (g/dl)	2.64±0.7	2.6±0.6	0.833	3.12±0.75	3.15±0.74	0.733
A:G ratio	1.46±0.33	1.51±0.3	0.363	1.1±0.2	1.1±0.21	0.683

Data are mean ± SD
