# Supplementary material for: Reference Intervals for Serum Protein Electrophoresis in the European Bison (Bison bonasus): A Comparison of Agarose Gel Electrophoresis and Capillary Zone Electrophoresis
Source: Vet Sci. 2026 Jun 30;13(7):644. doi: 10.3390/vetsci13070644 (PMC13418574; doi:10.3390/vetsci13070644)
Supplement: Supplementary file 1 [file vetsci-13-00644-s001.zip › Table S2.pdf]

Table S2: Differences between bison's captive and free-ranging for total protein and protein fractions for AGE and CZE in European bison (*Bison bonasus*) serum. Captive=85, Free=46

	AGE			CZE		
**Analyte**	**Captive**	**Free**	**P value**	**Captive**	**Free**	**P value**
Total protein (g/dl)	6.3±1	6.6±1.74	0.193	-	-	-
Albumin (%)	58.8±5.5	59.1±5.7	0.747	51±5.0	50.1±5	0.843
Albumin (g/dl)	3.7±0.69	3.9±0.95	0.226	3.21±0.63	3.31±0.63	0.358
α1-globulins (%)	6.5±1.5	5.9±1.6	0.048	3.93±1	3.53±1.1	0.726
α1-globulins (g/dl)	0.41±0.1	0.39±0.13	0.421	0.25±0.07	0.23±0.07	0.364
α2-globulins (%)	10.4±1.3	9.4±2	0.001	15.5±3.1	15.8±2.9	0.138
α2-globulins (g/dl)	0.66±0.12	0.62±0.17	0.109	1±0.27	1.02±0.28	0.207
β1-globulins (%)	6.89±1.19	6.7±1.12	0.072	6.6±1.05	6.5±1.07	0.055
β1-globulins (g/dl)	0.44±0.08	0.43±0.11	0.392	0.42±0.09	0.41±0.09	0.747
β2-globulins (%)	5.32±1.09	5.36±1.54	0.759	4.58±0.73	4.56±0.7	0.309
β2-globulins (g/dl)	0.34±0.09	0.33±0.11	0.454	0.29±0.09	0.3±0.08	0.881
γ-globulins (%)	11.7±3	13.4±2.9	0.002	19±3.6	18.3±3.3	0.000
γ-globulins (g/dl)	0.74±0.26	0.88±0.3	0.001	1.22±0.41	1.19±0.36	0.021
Total globulins (%)	40.9±5.3	40.9±5.7	0.944	48.8±5.8	48.6±5	0.944
Total globulin (g/dl)	2.57±0.54	2.72±0.81	0.354	3.11±0.75	3.14±0.74	0.415
A:G ratio	1.47±0.32	1.5±0.35	0.764	1.07±0.23	1.08±0.21	0.681

Data are mean ± SD
