# Supplementary material for: Reference Intervals for Serum Protein Electrophoresis in the European Bison (Bison bonasus): A Comparison of Agarose Gel Electrophoresis and Capillary Zone Electrophoresis
Source: Vet Sci. 2026 Jun 30;13(7):644. doi: 10.3390/vetsci13070644 (PMC13418574; doi:10.3390/vetsci13070644)
Supplement: Supplementary file 1 [file vetsci-13-00644-s001.zip › Table S3.pdf]

Table S3: Differences between age for total protein and protein fractions for AGE and CZE in European bison (*Bison bonasus*).

Calves= 28; Young= 49; Adults= 51

	AGE				CZE			
**Analyte**	**Calves**	**Young**	**Adults**	**P value**	**Calves**	**Young**	**Adults**	**P value**
Total protein (g/dl)	6.2±0.9	6.3±1	6.3±1.4	0.389	-	-	-	-
Albumin (%)	60.6±5.1	57.9±5.4	60.1±5.1	0.045	52.2±4.2	50.6±4.8	51.7±4.9	0.293
Albumin (g/dl)	3.8±0.8	3.7±0.7	3.8±0.9	0.271	3.26±0.61	3.18±0.53	3.31±0.74	0.555
α1-globulins (%)	6.22±1.4	7±1.7	5.7±1.2	0.002	3.9±1	4±1.2	3.6±0.8	0.046
α1-globulins (g/dl)	0.38±0.06	0.44±0.1	0.36±0.1	0.000	0.24±0.06	0.25±0.07	0.23±0.07	0.504
α2-globulins (%)	10.5±1.5	10.5±1.3	9.4±1.8	0.001	15.5±3.2	16.1±2.7	14.4±3	0.015
α2-globulins (g/dl)	0.65±0.1	0.66±0.13	0.59±0.14	0.009	0.96±0.24	1±0.25	0.93±0.28	0.260
β1-globulins (%)	7±1.1	7.2±1.1	6.7±1	0.067	6.6±1	6.6±0.8	6.3±0.95	0.063
β1-globulins (g/dl)	0.43±0.07	0.45±0.09	0.43±0.1	0.396	0.41±0.05	0.41±0.07	0.4±0.11	0.801
β2-globulins (%)	5±0.92	5.3±1	5.3±1.3	0.401	4.4±0.6	4.7±0.6	4.5±0.8	0.216
β2-globulins (g/dl)	0.31±0.07	0.33±0.1	0.33±0.11	0.575	0.27±0.05	0.3±0.08	0.3±0.11	0.517
γ-globulins (%)	10.4±2.6	12.2±2.9	12.8±2.6	0.001	17±2.5	18±3.1	19.6±3.4	0.003
γ-globulins (g/dl)	0.64±0.18	0.77±0.26	0.82±0.27	0.059	1.1±0.23	1.2±0.34	1.28±0.44	0.059
Total globulins (%)	39±4.9	42.1±5.4	39.9±5.1	0.025	17.4±5	49.5±4.8	48.3±5	0.151
Total globulins (g/dl)	2.4±0.34	2.67±0.57	2.52±0.59	0.195	2.94±0.47	3.13±0.47	3.14±0.87	0.059
A:G ratio	1.6±0.32	1.4±0.31	1.5±0.33	0.037	1.1±0.19	1±0.2	1.09±0.21	0.248

Data are mean ± SD
