# Supplementary material for: Reference Intervals for Serum Protein Electrophoresis in the European Bison (Bison bonasus): A Comparison of Agarose Gel Electrophoresis and Capillary Zone Electrophoresis
Source: Vet Sci. 2026 Jun 30;13(7):644. doi: 10.3390/vetsci13070644 (PMC13418574; doi:10.3390/vetsci13070644)
Supplement: Supplementary file 1 [file vetsci-13-00644-s001.zip › Table S4.pdf]

Table S4. Analysis of concordance between methods of electrophoresis for European bison (*Bison bonasus*) serum when data are expressed in percentage. Intercept, slope and residual standard deviation of Passing-Bablok regression as well as bias, lower and upper limit of agreement from de Bland-Altman analysis and their 95% confidence intervals (95% CI) from the comparisons between the AGE and CZE electrophoresis.

	Passing-Bablok regression			Bland-Altman plot		
**Fraction**	**Intercept (95% CI)**	**Slope (95% CI)**	**Residual standard deviation (95% CI)**	**Bias (95% CI)**	**Lower limit (95% CI)**	**Upper limit (95% CI)**
Albumin	1.9 (-2.8 to 6.9)	1.11 (1.01 to 1.2)	1.92 (-3.76 to 3.76)<br>Cusum test = 0.94	7.9 (7.4 to 8.34)	2.5 (1.68 to 3.31)	13.2 (12.4 to 14.1)
α1-globulins	-0.86 (-2.45 to 0.46)	1.8 (1.5 to 2.2)	0.84 (-1.7 to 1.7)<br>Cusum test = 0.11	2.5 (2.31 to 2.77)	-0.07 (-0.46 to 0.32)	5.16 (4.74 to 5.6)
α2-globulins	2.3 (0.76 to 3.5)	0.5 (0.42 to 0.61)	1.2 (-2.33 to 2.33)<br>Cusum test = 0.06	-5.3 (-5.7 to -4.9)	-9.9 (-10.5 to -9.2)	-0.76 (-1.45 to -0.07)
β1-globulins	0.08 (-0.99 to 0.96)	1 (0.91 to 1.22)	0.67 (-1.31 to 1.31)<br>Cusum test = 0.84	0.36 (0.2 to 0.53)	-1.48 (-1.76 to -1.2)	2.22 (1.94 to 2.5)
β2-globulins	-3.5 (-5.1 to -2.1)	21.92 (1.6 to 2.3)	0.52 (-1.02 to 1.02)<br>Cusum test = 0.13	0.75 (0.58 to 0.93)	-1.17 (-1.47 to -0.87)	2.68 (2.38 to 2.9)
γ-globulins	-3.95 (-5.37 to -2,59)	0.88 (0.8 to 0.96)	1.28 (-2.51 to 2.51)<br>Cusum test = 0.84	-6.4 (-6.69 to -6.05)	-10 (-10.6 to -9.5)	-2.74 (-3.29 to -2.2)
Total globulins	-11.8 (-16.9 to-7.3)	1.08 (0.98 to 1.19)	2.5 (-4.9 to 4.9)<br>Cusum test = 0.94	-7.8 (-8.42 to -7.2)	-14.7 (-15.7 to -13.6)	-0.94 (-1.98 to 0.08)
A:G ratio	-0.23 (-0.39 to -0.08)	1.6 (1.45 to 1.74)	0.11 (-0.22 to 0.22)<br>Cusum test = 0.30	0.41 (0.37 to 0.44)	0.02 (-0.04 to 0.08)	0.8 (0.74 to 0.86)
