# Supplementary material for: Reference Intervals for Serum Protein Electrophoresis in the European Bison (Bison bonasus): A Comparison of Agarose Gel Electrophoresis and Capillary Zone Electrophoresis
Source: Vet Sci. 2026 Jun 30;13(7):644. doi: 10.3390/vetsci13070644 (PMC13418574; doi:10.3390/vetsci13070644)
Supplement: Supplementary file 1 [file vetsci-13-00644-s001.zip › Table S5.pdf]

Table S5. Analysis of concordance between methods of electrophoresis for European bison (*Bison bonasus*) serum when data are expressed in g/dl. Intercept, slope and residual standard deviation of Passing-Bablok regression as well as bias, lower and upper limit of agreement from de Bland-Altman analysis and their 95% confidence intervals (95% CI) from the comparisons between the AGE and CZE electrophoresis.

	Passing-Bablok regression			Bland-Altman plot		
**Fraction**	**Intercept (95% CI)**	**Slope (95% CI)**	**Residual standard deviation (95% CI)**	**Bias (95% CI)**	**Lower limit (95% CI)**	**Upper limit (95% CI)**
Albumin	-0.46 (-0.64 to -0.28)	1.29 (1.24 to 1.35)	0.11 (-0.23 to 0.23)<br>Cusum test = 0.41	0.52 (0.48 to 0.56)	0.04 (-0.03 to 0.11)	0.99 (0.92 to 1.06)
α1-globulins	-0.04 (-0.11 to 0.03)	1.75 (1.46 to 2.06)	0.05 (-0.11 to 0.11)<br>Cusum test = 0.14	0.16 (0.15 to 0.18)	-0.01 (-0.04 to 0.004)	0.33 (0.31 to 0.36)
α2-globulins	0.16 (0.11 to 0.2)	0.49 (0.43 to 0.55)	0.07 (-0.14 to 0.14)<br>Cusum test = 0.09	-0.35 (-0.37 to -0.32)	-0.68 (-0.73 to -0.64)	0.008 (-0.06 to 0.04)
β1-globulins	-0.01 (-0.06 to 0.03)	1.1 (0.99 to 1.2)	0.038 (-0.07 to 0.07)<br>Cusum test = 0.94	0.02 (0.01 to 0.03)	-0.08 (-0.09 to -0.06)	0.13 (0.11 to 0.15)
β2-globulins	-0.1 (-0.12 to -0.03)	1.4 (1.25 to 1.6)	0.04 (-0.07 to 0.07)<br>Cusum test = 0.52	0.04 (0.03 to 0.05)	-0.06 (-0.08 to -0.05)	0.16 (0.14 to 0.17)
γ-globulins	-0.04 (-0.1 to 0.009)	0.7 (0.65 to 0.75)	0.09 (-0.17 to 0.17)<br>Cusum test = 0.70	-0.42 (-0.45 to -0.39)	-0.75 (-0.8 to -0.7)	-0.09 (-0.14 to -0.04)
Total globulins	0.07 (-0.08 to 0.21)	0.81 (0.77 to 0.86)	0.15 (-0.29 to 0.29)<br>Cusum test = 0.99	-0.53 (-0.57 to-0.49)	-1.02 (-1.1 to-0.95)	-0.03 (-0.11 to 0.04)
