# Supplementary material for: Reference Intervals for Serum Protein Electrophoresis in the European Bison (Bison bonasus): A Comparison of Agarose Gel Electrophoresis and Capillary Zone Electrophoresis
Source: Vet Sci. 2026 Jun 30;13(7):644. doi: 10.3390/vetsci13070644 (PMC13418574; doi:10.3390/vetsci13070644)
Supplement: Supplementary file 1 [file vetsci-13-00644-s001.zip › Table S6.pdf]

Table S6. Concordance correlation analysis between AGE and CZE electrophoresis. Lin's concordance correlation coefficients (r_{ccc}) with 95% confidence intervals (95% CI), precision (Pearson p) and accuracy (bias correction factor, C_b) are reported.

**Measured variables**	**Lin's r_{ccc} (95% CI)**	**Precision p**	**Accuracy C_b**
Albumin (%)	0.409 (0.34 to 0.48)	0.869	0.471
Albumin (g/dl)	0.751 (0.7 to 0.8)	0.9743	0.772
α1-globulins (%)	0.170 (0.11 to 0.22)	0.527	0.322
α1-globulins (g/dl)	0.2 (0.14 to 0.26)	0.576	0.351
α2-globulins (%)	0.159 (0.11 to 0,20)	0.649	0.244
α2-globulins (g/dl)	0.296 (0.24 to 0.35)	0.838	0.354
β1-globulins (%)	0.557 (0.43 to 0.66)	0.592	0.941
β1-globulins (g/dl)	0.805 (0.73 to 0.86)	0.832	0.967
β2-globulins (%)	0.427 (0.33 to 0.52)	0.64	0.671
β2-globulins (g/dl)	0.69 (0.61 to 0.77)	0.81	0.861
γ-globulins (%)	0.291 (0.23 to 0.35)	0.85	0.346
γ-globulins (g/dl)	0.501 (0.43 to 0.57)	0.926	0.542
Total globulins (%)	0.389 (0.31 to 0.46)	0.793	0.491
Total globulins (g/dl)	0.738 (0.68 to 0.79)	0.957	0.771
A:G ratio	0.358 (0.3 to 0.42)	0.805	0.444

Values of Lin's $r_{ccc} < 0.5$ were considered unacceptable
